# The Struggle for Existence Becomes General

**Published:** 1878-01

**Authors:** 


### EDITORIAL.

### The Struggle For Existence Becomes General.

The "struggle," has heretofore been confined to the rank and file of the medical profession while the commanding and subordinate officers have been exempt from attack and have for the most part looked on and noted the progress of the war. but now it is changed. The lights of the profession, the guides are being attacked. It is charged that they Advertise very much like the Quacks. The "Code"—the organic law—the standard of morals is charged with defects,—as operating unfairly and opressively. So far as suppression of quackery is concerned, it is charged as being a failure, and the President of the American Medical Association, Dr. Simons tells the members "that it requires revision."

Art. 1st of the code says "it is derogatory to the dignity of the profession to resort to public advertisements, or private cards, or hand-bills inviting the attention of individuals affected with particular diseases, publicly offering advice and medicine to the poor gratis or promising radical cures; or to publish cases and operations in the daily prints, or to suffer such publications to be made; to invite laymen to be present at operations, to boast of cures and remedies, to adduce certificates of skill and success, or to perform any other similar acts. These are the ordinary practices of empirics, and highly reprehensible in a regular physician."

Dr. Chenoweth points out to the medical society of central Illinois how the code is defective and how its provisions are ignored and avoided. Showing how medical colleges and their professors *do* advertise in Illinois at least, perhaps sometimes in Ohio and New York, and how specialists do disregard the medical code every where and still try to retain fellowship with respectable physicians, He says :

"The first clause aims to prevent specialists from resorting to public advertisements and the American Medical Association has decreed that they shall not advertise in medical journals, so that an advertisement of a specialty in a medical journal is just as much an infringement as the same would be in a newspaper. How then do specialists advertise, if at all? A common mode is to establish a private hospital, place it under the care of a religious society, and advertise themselves as consulting physicians. This is done in nearly all of our large cities, and while there is a deal of seeming modesty in it, it is so effectual that he is blind who cannot see it. Another mode quite as effectual is by reprinting essays which have been contributed to medical journals, and sending them to physicians and druggists all over the country. But whatever the mode adopted there is no question of the fact that they do advertise, and so effectually as to be known outside of the profession almost as well as in the profession.

The next clause forbids "publicly offering advice and medicine to the poor gratis." This is almost a dead letter because of public charities, and because of the call for patients at the clinics of the professors in our medical colleges. In 1875 the editor of a prominent journal published in the city of New York said. "when a new hospital or dispensary is started, it is not an uncommon thing to see placards upon the fences announcing the fact, and a general invitation to the public to avail themselves of the privilege of being treated gratis." And the fact that treatment can be had free at college clinics is so well advertised that most of us suffer the consequences whenever it becomes known that we propose a surgical operation. Very recently I contracted to operate on a lad for stone in the bladder, and appointed a day for the operation, but before the time arrived the father of the boy was induced to take him to a neighboring city, where the stone was removed without charge.

The third clause prohibits from promising radical cures. Promises of this kind are seldom made directly, but there is a constant disposition on the part of writers in medical journals, and of medical books, to so state their cases as to promise by implication the cure of like cases.

The fourth aims to prevent the publication of cases and operations in the daily papers. It is a much more difficult matter to avoid wilfully violating this rule to-day than ever before. As an illustration, Senator Ferry, of Michigan, was taken sick just after the close of the last session of the American Medical Association, and the attention rendered to him by Dr. J. H. Hollister, of Chicago, was as well known in every city and town in the United States as it was by Senator Ferry's household in Detroit—having been announced in the associated press dispatches. Last year when Senator Blaine was sick, daily, and almost every hour, his physician gave reports of his condition. True, he was an irregular practitioner, but had he been as regular as the most scrupulous member of this society his name would have been in every newspaper in the land The not over-nice physician, whose reputation is insufficient to command the practice of the great, and who wishes to advertise can do so without laying himself liable, and that he does we have every necessary proof. A medical journal of general circulation in the West said, not long since, "To-day physicians advertise practically without limit ar censure, provided they use some ingenuity in appearing in the text columns of the newspaper, and not in those devoted to advertisements; and provided, also, that they have some little local or general influence, and do not belong to the unhappy army of professional strugglers for bread, who, if under the pressure of want violate the law, are remorsely crucified, and publicly held up as a warning and example.

The fifth clause prohibits inviting laymen to be present at operations. How far this is obeyed, generally, I do not know, but I have not heard of an instance where they were kept away if they desired to be present.

The sixth clause denounces boasting of cures and remedies—alluding, doubtless, to conversation as well as advertising in the press. This is so common an offense that he who is not guilty should be looked upon as a paragon.

The complacency with which Dr. Gross speaks of the amount and kind of of work he bestowed on surgery, and of the conscientious manner in which he has taught surgery, is an exhibition of self-confidence which in an inferior man, or one whose work did not correspond to the estimate placed on it by himself, would be regarded as self-praise, and has probably been the innocent cause of not a little boasting by inferior men in regard to their practice. The Dr. says: "I have been unceasingly devoted to the duties of an arduous practice, both private and public; to the study of the great masters of the art and science of medicine and surgery; and to the composition of various monographs having a direct bearing upon a number of the subjects discussed in these volumes. The work therefore should be regarded as embodying the results of a large personal, if not ripe, experience; of extensive reading; and much reflection; in a word, as exhibiting surgery as I myself understand it, and as I have for so many years conscientiously taught it."

In the annual address before the New York Academy of Medicine, 1858, J. Marion Sims claimed that the use of the silver wire suture originated with him, and that it is "the great surgical achievement of the nineteenth century." The society not only accepted his statements but ordered the address to be printed in pamphlet form for distribution. Every body did not agree with him. A celebrated physician, in review of the address, said that "were it not in the black and white of a printed pamphlet published 'by order,' etc, and with all its *ad captandum* paraphernalia of pictures and staring capitals" he would "try to regard its various peculiarities as the harmless frothiness of an after dinner effusion, easily accounted for and as readily forgotten," and further stigmatised it as a "tissue of adulation and self-glorification." If Sims violated the spirit of the Code it did not prevent his election as president of the American Medical Association. Spencer Wells *par excellence* the ovariotomist, does not hesitate to tabulate the result of variotomy for the last nine years in some of the large general hospitals in London, and show a very favorable result in the Samaritan (his) hospital as compared with others. True, he offers as a reason for making these results public, "that the larger hospitals would be encouraged to do all that could be done by efficient sanitary precautions to assist in insuring greater success in the future. * There is no such useful stimulus as a little wholesale rivalry." If any one else had made the comparison we would have no right to charge boasting to him, but as he did it, and showed a success from two to three times greater for himself, it may be that a little just pride excited the act. Though he was careful to be judged of by the results of his operations, obeying the scripture injunction, "let not him that girdeth on his harness boast himself as he that putteth it off.

Seventh, to adduce certificates of skill and success. This differs but little from the last, since reports of cases and reference to parties is almost identical with certificates from the parties, especially if permission has been obtained to refer to them—a thing frequently done when boasting of cures.

The last prohibitition is intended to cover like acts not specified. As this is a general provision it has but little binding force, being subject to different interpretations according to taste or inclination. That we do not exaggerate in reference to violations of the Code, will be apparent to any one who will notice the phillipics against members of the profession, found in medical journals, for irregularities in practice. Many of them, doubtless, are intended to

> "Compound for sins they're inclined to
> By damning those they have no mind to."

But the sins exist or there would not be so many notices of it. But if the journals ignored the fact, the troubles arising in County and State Societies show plainly enough that there are many violations of the Code, or very many persons who are straining at gnats. Many of these difficulties doubtless arise from honest difference of opinion. Certain persons, believing that it is right to advertise undertake to follow the dictates of their own judgment; or knowing that advertising is done extensively by parties of reputation, claim that as these persons are not censured their course is approved—silence being construed into endorsement. For instance, about ten years after the adoption of the Code of Ethics by the American Medical Association, Dr. Draper, the accomplished Professor of Chemistry of the medical department of the New York University, published a very elaborate and elegant circular containing his testimonials as a physician and surgeon. I do not know what action, if any, was taken by the association in regard to it; but his interpretation of, or contempt for, the Code doubtless influenced others to a like course."

Our Illinois friend has stated this "code" question so truthfully that we have indulged in a liberal quotation mostly because he passes over the obscure and unimportant advertiser and shows how the very best manage to avoid and disregard the very laws they have been most active to make and inforce. The Editor of the New York Record points out how wise and good men in New York advertise (gratuitously), and yet whose professional standing is little if at all lowered. Possibly our code *is* useless for good and should be amended and enforced or discarded altogether. There are a great many physicians who greatly desire to advertise but are not yet in position to do so satisfactorily, and are restrained. Men who have nothing to advertise do advertise it extensively and on this account we mistrust that the physicians who are competent, honest and truthful, have no desire or willingness to enter the long list of professional vagabonds who fill every corner with the nauseating accounts of themselves. How can men anxious for distinction think of obtaining it, or anything but infamy by self-adulation and how can physicians of lower grade looking only for the rations of professional notoriety expect to gain this end by any of the various expedients complained of.

The honest unpretending non-advertising intelligent physician *only* can have reputation, honor and distinction. Whosoever thinketh up some other way, the same is a thief and a robber.

